# Tetrodotoxin, an Extremely Potent Marine Neurotoxin: Distribution, Toxicity, Origin and Therapeutical Uses

**DOI:** 10.3390/md13106384

**Published:** 2015-10-19

**Authors:** Jorge Lago, Laura P. Rodríguez, Lucía Blanco, Juan Manuel Vieites, Ana G. Cabado

**Affiliations:** Food Safety Division, ANFACO-CECOPESCA, Carretera del Colegio Universitario 16, 36310 Vigo, Spain; E-Mails: jlago@anfaco.es (J.L.); lperez@anfaco.es (L.P.R.); lucia@anfaco.es (L.B.); direccion@anfaco.es (J.M.V.)

**Keywords:** tetrodotoxin, toxicity, origin, distribution, medical uses

## Abstract

Tetrodotoxin (TTX) is a potent neurotoxin responsible for many human intoxications and fatalities each year. The origin of TTX is unknown, but in the pufferfish, it seems to be produced by endosymbiotic bacteria that often seem to be passed down the food chain. The ingestion of contaminated pufferfish, considered the most delicious fish in Japan, is the usual route of toxicity. This neurotoxin, reported as a threat to human health in Asian countries, has spread to the Pacific and Mediterranean, due to the increase of temperature waters worldwide. TTX, for which there is no known antidote, inhibits sodium channel producing heart failure in many cases and consequently death. In Japan, a regulatory limit of 2 mg eq TTX/kg was established, although the restaurant preparation of “fugu” is strictly controlled by law and only chefs qualified are allowed to prepare the fish. Due to its paralysis effect, this neurotoxin could be used in the medical field as an analgesic to treat some cancer pains.

## 1. Introduction

**Figure 1:**
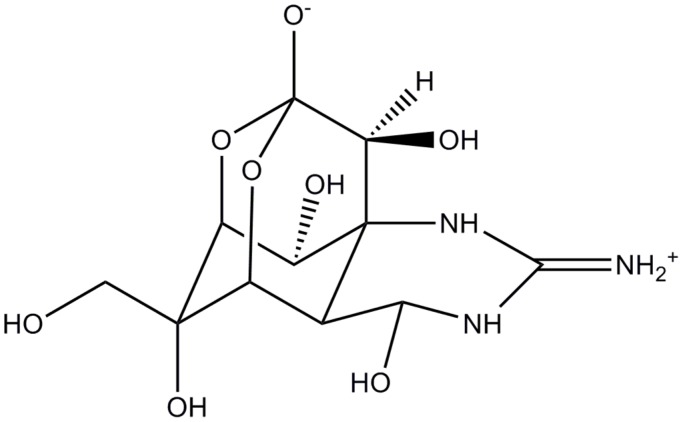
Chemical structure of tetrodotoxin (TTX).

In July 1894, Dr. Yoshizumi Tahara presented the poison isolated from aqueous extract of ovaries of globefish at the monthly meeting of the Pharmaceutical Society of Japan. Later, he established an improved method for extraction and purification suitable for large-scale production of the poison. Finally, in 1909, he confirmed that globefish contains only one toxic substance and named it tetrodotoxin (Figure 1) due to the name of the family of pufferfish from which it was first isolated, *Tetraodontidae* [1].

At first, it was believed that TTX was present exclusively in pufferfish of the family *Tetraodontidae*, and it was controversial whether TTX in the fish was endogenous (produced by the pufferfish itself) or exogenous (taken from the outside and accumulated). In 1964, this toxin was unexpectedly detected in the Californian newt *Taricha torosa* [2] breaking the above belief. Since then, TTX has been detected in both marine [3,4,5,6,7,8,9] and terrestrial species [10,11,12,13]. Moreover, the TTX intoxication of the trumpet shell following the ingestion of toxic starfish [7], TTX production by marine bacteria [14], the facts that pufferfish become non-toxic when artificially reared with non-toxic diets [15,16,17] and that such non-toxic pufferfish become toxic when fed TTX-containing diets [18,19,20], have pointed out that the main mechanism of TTX accumulation in pufferfish is the food chain, consisting of several steps and starting with marine bacteria as a primary source of TTX. In marine pufferfish species, toxicity is generally high in the liver and ovary, whereas in freshwater species, toxicity is higher in the skin.

Although TTX-bearers are typical of warm waters and it was regarded as a problem confined to Asian countries, recent studies report the possible migration of these toxic species from the Red Sea to the Mediterranean Sea through the Suez Canal [21,22,23].

TTX, one of the most powerful neurotoxins known, it is about 1200 times more toxic to humans than cyanide and it has no known antidote. This toxin binds to the sodium channels of the excitable tissues in the human body (muscles and nerves) and the inhibition of sodium ions through the channels effectively immobilises these tissues [24]. The severity of the symptoms induced by the TTX is dose dependent [25]. The symptoms included tingling of the tongue and lips, headache, vomiting, muscle weakness, ataxia and even death due to respiratory and/or heart failure [26].

TTX is responsible for 30–50 cases of intoxications occurred every year [26]. In Japan, a regulatory limit of 2 mg equivalent of (eq) TTX/kg was established for TTX, while in Europe, this compound, considered an emerging toxin, is still not regulated. However, according to the current EU legislation, fish belonging to the poisonous family of fish *Tetraodontidae* or products derived from it must not be placed on the European markets [27,28].

Because TTX blocks voltage-gated sodium channel and causes paralysis, it can also be applied as a potential pain relief and some researchers are trying to make use of the analgesic activity of TTX to treat various types of pains such as severe cancer pain [29,30,31], or to help in reducing cue-induced increases in heroin craving and associated anxiety [32].

In the present paper, the detailed origin and distribution in nature, the toxicity and mechanism of action and the different medical uses of TTX are thoroughly described.

## 2. Distribution in Nature

TTX is a non-protein, low molecular weight neurotoxin first believed to be present only in pufferfish of the family *Tetraodontidae*. However, it was detected in a disparate array of phylogenetically unrelated terrestrial and aquatic organisms: a dinoflagellate, red calcareous algae, arthropods, echinoderms, molluscs, worms, newts and frogs. TTX has also been identified in sediments from marine and freshwater environments [33].

Regardless of the abundant research, there is uncertainty about the biosynthesis or biological origin of TTX as well as its ecological function. It is supposed that arginine is the precursor moiety for TTX production within the organism [34].

The presence of TTX in such a wide diverse array of taxa has been taken as an indication that the ultimate origin of TTX in metazoans must be exogenous. Indeed, there is good evidence that uptake of bacteria producing TTX is an important element of TTX toxicity in marine metazoans that present this toxin (reviewed in [35], cited in [36]). However, this model has been questioned in regards to the TTX that contain terrestrial taxa [33,37,38,39]. On the contrary, endogenous production of TTX means that it is derived from elements of the diet.

It was reported that bacteria were responsible for producing TTX. In fact, many bacteria have been isolated from marine organisms, although, the TTX levels produced by these bacteria seem too low to account for the concentrations found in toxic organisms. In addition, there are not specific techniques to prove that the TTX has a microbial origin [40].

### 2.1. Aquatic Animals

Endocellular symbiotic bacteria have been proposed as a possible source of eukaryotic TTX by means of an exogenous pathway. Many of the TTX-containing marine species such as pufferfish [41] and xanthid crabs [14] have been found to harbour TTX producing bacteria within their microbiome as determined by chemical analysis and toxicity assay of media inoculated with isolated bacteria [41,42]. Such TTX-producing bacteria include species identified from the following genera: *Actinomyces*, *Aeromonas*, *Alteromonas*, *Bacillus*, *Pseudomonas* and *Vibrio*. Some of them were isolated in particular from a determined specie; for instance, *Vibrio alginolyticus* was isolated from starfish, *Vibrio* spp. and *Aeromonas* from the pufferfish and *Vibrio* and *Pseudomonas* spp. from gastropods [34]. Both *Bacillus* and *Pseudomonas* species from the venom-producing posterior salivary gland of blue-ringed octopus were found to produce TTX as confirmed by mouse toxicity assay [43].

The statement that TTX has an exogenous origin in fresh water and marine organisms is supported by several studies. An exogenous origin for TTX is suspected for certain toxic crabs which feed on small gastropods known to contain TTX and on marine sediments containing TTX-positive bacteria. Therefore, it is assumed that crabs obtain TTX from the food chain. Toxicity in snails, on which crabs feed, suggests that there is a regional variation that subsequently correlates with the toxicity of crabs. This would imply an exogenous origin of TTX in both snails and the crabs, higher up the food chain [41].

In a recent report, samples of the grey side-gilled sea slug *Pleurobranchaea maculata* were collected from 10 populations around New Zealand and TTX levels assessed using liquid chromatography-mass spectrometry (LC-MS). This study shows that the occurrence of TTX may involve symbiotic TTX-producing bacteria, though the possibility that TTX is accumulated through the food chain or produced endogenously cannot be fully excluded [40]. These sea slugs become non-toxic when they are fed on a TTX-free diet [41]. A small gastropod, *Umborium suturale*, a known progenitor of TTX and anhydroTTX, was isolated from the digestive gland of starfish suggesting an exogenous origin for TTX in this starfish.

Nevertheless, an evidence for a TTX endogenous origin for gastropods is that TTX was higher in the muscle than in the digestive gland of the snail *Natica lineata* and the edible gastropod, *Polinices didyma*. Of course, in the case of the gastropods, it is possible that after initial ingestion of TTX, the toxin could have then migrated and remained in tissue compartments outside of the digestive region [34].

#### Pufferfish

The amount of TTX accumulated in pufferfish depends on the species and varies among organs in different seasons but it is concentrated mainly in ovary, liver, and other body parts, as the intestine. It was reported that juvenile cultivated pufferfish in aquaria or in cages suspended above the sea floor become non-toxic over time in captivity. They became toxic when they were grown in open water again or were fed with toxic puffer livers, whereas certain other species of pufferfish did not become toxic. For instance, pufferfish, *Takifugu rubripes* becomes non-toxic when it is fed on a TTX-free diet. Also, when pufferfish were fed with a TTX-containing diet their toxicity increased significantly [41]. These experimental findings suggested that TTX can be acquired and accumulated from the food chain and that certain species of pufferfish may possess a functional ability to store or eliminate this toxin. Relevant evidence for this possibility is the observation of saturation uptake kinetics for TTX revealed by tissue of pufferfish liver which is indicative of a carrier-mediated membrane transport system for the toxin [44]. In fact, a binding protein for TTX as well as for saxitoxin (STX), another neurotoxin that blocks sodium channels, present in the plasma of pufferfish, may represent a possible mechanism for tissue delivery [45]. It has also been demonstrated that the source of TTX in pufferfish is an endosymbiotic bacteria that naturally inhabits the gut of the animal. It could be that pufferfish initially acquire the TTX producing bacteria via the food web and that these bacteria then persist in the fish.

Thus, available evidence is consistent with the interpretation that pufferfish and certain other marine species accumulate TTX directly from marine bacteria that synthesize this toxin. Alternatively, TTX storage by a given species may occur by numerous pathways of dietary transfer through a complex community food chain involving bacteria, plankton, invertebrates and fish [16]. In addition, since TTX is widely distributed among a wide range of species, it was suggested that this toxin has an exogenous microbial origin, rather than being produced by pufferfish *per se*. At least 12 different species of TTX-producing bacteria have been isolated from various pufferfish tissues including the skin, intestine, ovaries, and liver [41,42]. A newly TTX-producing bacterium, *Raoultella terrigena*, was isolated from the intestines of a local toxic pufferfish *Takifugu niphoble*. The TTX production ability of this strain was investigated and the 16S–23S rDNA ITS region was sequenced. The toxicity of the strain was confirmed by mouse bioassay, ELISA and mass spectrometry (MALDI-TOF). These outcomes reiterate that the TTX found in pufferfish was likely produced by the associated bacteria among a diversity of bacterial species [41]. An interesting discussion regarding how bacteria could produce TTX under certain physiological parameters was recently reported by Jal and Khora [46]. The concept of multiple horizontal gene transfer and synergistic production of secondary metabolites could be possible in the case of TTX-producing bacteria [46].

Table 1 represents most of the marine organisms, including invertebrates and vertebrates, as different species of pufferfish, in which bacteria were identified as TTX producers. Other TTX-producing bacteria and the host organisms are reviewed in Jal and Khora [46].

**Table 1:** TTX-producing bacteria isolated from several marine organisms.

Organisms	Species	Bacteria	Reference
Red algae	*Jania* sp.	*Vibrio*, *Alteromonas* and *Shewanella*	[47]
Crustacean: Copepods (Parasites of Pufferfish)	*Pseudocaligus fugu* and *Taeniacanthus* sp.	*Roseobacter*	[48]
Crustacean: xanthid crab	*Atergatis floridus*	*Vibrio* strain	[14]
Mollusc gastropod	*Niotha Clathrata*	*Vibrio* and *Pseudomonas*	[49]
Mollusc cephalopod	*Octopus maculosus*	*Bacillus*, *Pseudomonas alteromonas* and *Vibrio* spp.	[43]
Echinoderms: starfish	*Astropecten polyacanthus*	*Vibrio alginolyticus*	[50]
Vertebrates: pufferfish	*Takifugu snyderi*	*Vibrio* strain	[51]
Pufferfish	*Takifugu obscurus*	*Aeromonas*	[52]
Pufferfish	*Fugu poecilonotus*	*Vibrio*, *Alteromonas* and *Shewanella*	[47]
Pufferfish	*Takifugu niphobles*	*Raoultella terrigena*	[41]
Pufferfish	*Fugu obscurus*	*Bacillus* spp.	[53]
Pufferfish	*Fugu rubripes*	*Bacillus* and *Actinomycetes*	[54]
Pufferfish	*Chelonodon patoca*	*Microbacterium arabinogalactanolyticum*, *Serratia marcescens*, *Vibrio alginolyticus*	[55]
Pufferfish	*Fugu vermicularis radiatus*	*Vibrio* strain *LM-1*	[56]

### 2.2. Terrestrial Animals

Evidence supporting endogenous production of TTX has been obtained predominately from terrestrial organisms although the origin of this toxin in these species is very controversial [40]. TTX in terrestrial animals is limited to Amphibia as newts, toads and frogs [34].

Some authors argue that origin of TTX in terrestrial animals is endogenous because this toxin has a role in defence [34]. In particular, the levels of TTX and 6-epiTTX in newts are higher in the skin rather than in the liver, supposedly TTX is biosynthetically produced by the creature as a protection mechanism for predators.

It was suggested that the degree of coloration of frogs belonging to the *Brachycephalus* family can be related to its toxicity. The authors found the highest TTX levels in the skin followed by liver and ovaries of bright yellow frogs. However, cryptic coloration frog was found to be non-toxic. Interestingly, the “poison dart” frogs are brightly colored and are well known for high toxicity, though this derives not only from TTX. Then, the bright coloration protects these frogs from predators who instinctively avoid them. This fact strongly suggests that toxic frogs naturally synthesise the toxin, although, studies have not been performed to definitively corroborate this theory [34].

In frogs of the genus *Atelopus*, captive-raised individuals do not possess TTX, suggesting a dietary or other environmentally dependent origin of toxicity [33]. In captivity, TTX levels in the terrestrial newt, *Taricha granulosa*, lethal to almost all potential predators, increase with a TTX-free diet. Also, when it is induced to release TTX from their skin by electrical stimulation, regeneration of TTX apparently by secretion occurs within nine months, whereas captive-raised *Atetopus* toads lose TTX-toxicity [36]. Additionally, authors did not find evidence of bacterial symbionts (mtDNA signatures) in TTX-laden organs of *T. granulosa*. Although individual specimens of the newt *T. granulosa* can contain up to the equivalent of 6 mg of TTX, efforts to isolate TTX-producing bacteria from them have so far been unsuccessful [33,40,57]. Using PCR primers that specifically amplify 16S rRNA genes of bacteria, tissues from newts, *T. granulosa*, were examined for the presence of bacteria which may produce TTX. No amplification of bacterial DNA was seen in samples taken from skin, liver, gonads or eggs. Amplification of bacterial DNA was only seen in samples taken from newt intestines, a tissue with low concentrations of TTX. These results indicate that symbiotic bacteria are unlikely to be the source of TTX in newts [33]. However, researchers have found that the newt *Notophthalmus viridescens* becomes non-toxic when it is fed a TTX-free diet suggesting, that at least in this case, TTX has an exogenous origin [34].

Nevertheless, since many bacteria cannot be cultured by traditional methods, the absence of a cultivable isolate does not necessarily rule out a bacterial origin. More puzzling is the reported failure to detect incorporation of radioactivity into the TTX pool of newts that were fed likely carbon precursor molecules such as ^14^C-labeled arginine or glucose [57].

Taking into account the available information, it seems that some amphibians could acquire TTX from dietary sources while others could use endogenous mechanisms for toxin production.

### 2.3. Origin of STX vs. TTX

Similar to TTX, there is an interest in the biological origin of STX. In freshwaters, this toxin is produced by prokaryotic cyanobacteria while in marine waters it is associated with eukaryotic dinoflagellates. However, several studies suggest that STX is not produced by dinoflagellates themselves, but by cocultured bacteria. These authors show that genes required for STX synthesis are encoded in the nuclear genomes of dinoflagellates and that the dinoflagellate transcripts of sxtA have the same domain structure as the cyanobacterial sxtA genes. These results show very good agreement between the presence of sxtA and STX synthesis, except in three strains of *Alexandrium tamarense*, where they did not detect the toxin. They conclude that different genes in the sxt pathway may have separate origins in dinoflagellates [58].

Considering the biological origin of STX, a gene cluster (sxt) consisting of up to 26 genes that participate in the biosynthesis of STX, was identified. The sxt gene cluster has so far been found in five species of STX-producing cyanobacteria and in the nuclear genome of five species of dinoflagellates. Microbial production of STX is thus the accepted origin of STX subsequently transferred among various invertebrate and vertebrate species in the aquatic freshwater and marine food chains. In a recent report, the occurrence of the dinoflagellate *Prorocentrum minimum*, was linked to the presence of TTX in bivalves [59]. However, the dinoflagellate *A. tamarense* is the only STX-producing species that has been reported to also produce TTX in culture [57,60]. This suggests that biosynthetic genes for production of STX and TTX evolved largely independently in nature but leaves open the possibility that STX and TTX may share precursor molecules and some biosynthetic enzymes.

Moreover, the strong conservation in the sxt cluster, under radical changes in environmental conditions and organism diversity, shows that it has continued to play an important adaptive role in some cyanobacteria [57,61].

In conclusion, more than one biosynthetic mechanism for TTX production could have evolved as convergent in nature, taking into account the lack of an obvious biological source of TTX common to marine *vs.* terrestrial animal species. A likely scenario is that synthetic genes for TTX have transferred across species in evolution. A recent study proposed that origin of TTX may be due to any of the following combinations: exogenous, endogenous or by the symbiotic association among the animals acquiring it and the microorganisms that are reported produce it [46]. These questions emphasize the importance for future research of identifying natural biosynthetic genes for TTX. Despite all of these assumptions, the exact origin and pathway for the synthesis and bio-transfer of TTX is not yet fully known and requires further investigation. It is of high importance and an open question to gain more insights into the spread of TTX in both prokaryotic and eukaryotic systems as well as to elucidate the genes and enzymatic pathways responsible for the biosynthesis of TTX in bacteria.

## 3. Toxicity and Mechanism of Action

The mechanism of toxicity of TTX has been investigated in different animal models [62,63,64]. TTX acts by blockage of the sodium channels and reduces the membrane excitability of vital tissues, of the heart myocytes, skeletal muscles, and the central and peripheral nervous systems [65,66] resulting in the occurrence of typical symptoms and even death in the most severe cases [67]. A gradation of the intoxication severity, based on symptomatology, was established by Fukuda and Tani in 1941 (Table 2) [34,68].

The toxicity of TTX was investigated in mice and rabbits by some researchers. The median lethal doses (LD50) obtained in mice were 10.7, 12.5, 532 µg/kg for intraperitoneal (i.p.), subcutaneous (s.c.) and intragastric (i.g.) administration of toxin, respectively. During this study it was found that the male mice were more sensitive to TTX. On the other hand, the minimal lethal dose (MLD) obtained in rabbits were 5.3 and 3.1 µg/kg while the lethal doses (LD) were 5.8, 3.8 µg/kg for both intramuscular (i.m.) and intravenous (i.v.) administration, respectively. Moreover, the symptoms in both animal species were described and the results obtained indicated that TTX was found to be about 50 times less toxic to mice via oral administration than that via i.p. injection [69].

**Table 2:** Symptoms of TTX intoxication depending on the grade.

Grade	Symptoms
1	Neuromuscular symptoms (paresthesia around the mouth, headache, diaphoresis, pupillary constriction) and mild gastrointestinal symptoms (nausea, vomiting, hypersalivation, hyperemesis, hematemesis, hypermotility, diarrhea, abdominal pain).
2	Paresthesia spreading to the trunk and extremities, early motor paralysis and lack of coordination.
3	Increased neuromuscular symptoms (dysarthria, dysphagia, aphagia, lethargy, incoordination, ataxia, floating sensation, cranial nerve palsies, muscular fasciculations) cardiovascular/pulmonary symptoms (hypotension or hypertension, vasomotor blockade, cardiac arrhythmias including sinus bradycardia, asystole, tachycardia, and atrioventricular node conduction abnormalities; cianosis, pallor, dyspnea); dermatologic symptoms (exfoliative dermatitis, petechiae, blistering) hypotension, and aphonia.
4	Impaired conscious state, respiratory paralysis, severe hypotension, and cardiac arrhythmia.

People intoxicated started to present symptoms within 30 min to 6 h after ingestion, with fully recovery usually in 24 h [70,71]. Some of the symptoms induced by the TTX are headache, diaphoresis, body numbness, dysarthria, dysphagia, nausea, vomiting, abdominal pain, generalized malaise, weakness, and lack of coordination and, in more severe cases, hypotension, cardiac arrhythmias, muscle paralysis, and cranial nerve dysfunction may develop. Death can occur in most critical cases due to respiratory failure and cardiovascular collapse as early as 17 min after ingestion [72].

## 4. Human Cases of Intoxication

### 4.1. Asian Countries

More than 100 cases of human intoxication were reported in Taiwan from 1998–2008 [73]. The highest concentration of TTX in pufferfish was found in the viscera (gonads, liver and intestine) and skin [74]. In November, 1998, a food poisoning incident due to ingestion of roe of *Takifugu oblongus* occurred affecting eight people including five deaths. Their symptoms resembled those caused by TTX. Twenty-two specimens of *T. oblongus* collected from the seashore adjacent to the concerned poisoning area, showed a high level of toxicity in the ovary (24.5–323.8 MU/g), though the toxicity levels of the other tissues, skin, muscle, liver, testis, and the viscera (except liver), were relatively low (<2–21.3 MU/g). The toxin purified by HPLC analysis from the *T. oblongus* specimens, was TTX [6]. On 18 April 2002, 37 patients (male 19, female 18) with manifestations of pufferfish poisoning were admitted to Khulna Medical College Hospital, Khulna, Bangladesh with a history of consumption of pufferfish (40–400 g). Symptoms observed were peri-oral paresthesia, weakness of both lower limbs, paresthesia all over the body, headache, difficulty in respiration, nausea and vomiting, blurring of vision, and vertigo. Twenty-two patients developed ascending paralysis of the limbs, and the respiratory muscle were involved in other patients. Fourteen patients had manifestations within 30 min of ingestion. Out of these 37 cases, eight patients died within five hours of post-ingestion. The cause of death in all these patients was respiratory muscle paralysis leading to respiratory failure [75].

Another outbreak occurred in the village of Maiskhal, in southeastern Bangladesh during October 2008. Of the nine persons who ingested the pufferfish, six showed development of symptoms. Five of these persons became severely ill and were taken to the local hospital. The most common symptoms were vomiting and diarrhea followed by paresis of the limbs and a tingling sensation. The median duration between consumption of the pufferfish eggs and illness onset was three hours. The five persons who consumed more than 20 g of the pufferfish egg showed development of severe illness. Two of these persons died and the other three were treated with neostigmine and atropine at the hospital and survived [76].

In other Asiatic regions several outbreaks were produced. In Chon Buri, in the eastern coast of Thailand, 71 persons were intoxicated due to the consumption of the crab *Carcinoscorpius rotundicauda*. Paresthesia, vertigo, weakness, respiratory paralysis, altered consciousness with unreactive dilated pupils, nausea and vomiting were some of the symptoms found in patients. During this outbreak, nineteen patients required artificial ventilation and two died [77].

One 48-year-old man died in Nagasaki, Japan, in October of 1996 due to the ingestion of the pufferfish *Takifugu poecilonotus*. One hour after the ingestion he began to suffer from numbness in hands and limbs, followed by cyanosis and respiratory failure. He died during the following hour [78].

### 4.2. Other Continents

#### 4.2.1. America

TTX was regarded until recently as a problem confined to Asian countries, but nowadays the problem is emerging as a threat to regions previously considered as safe. In 1986, a 45-year-old man in Hawaii ate the liver of the toxic porcupinefish *Diodon hystrix* and developed mild tetrodotoxication consisting of hyperemesis, bradycardia, hypotension, generalized numbness, and a generalized paresis. He was treated with atropine, normal saline IV infusions, nasogastric suction, and oxygen, and he recovered after six days [79].

In 2007, two individuals were intoxicated with TTX poisoning after ingesting pufferfish belonged to the family *Tetraodontidae* purchased in Chicago. TTX was detected at high levels in the ingested meal [80]. Another two patients with symptoms suggestive of TTX poisoning went to the Hennepin County Medical Center Emergency Department in Minneapolis, Minnesota on 13 June 2014 after the ingestion of dried pufferfish purchased in New York City. The pufferfish was identified as *Lagocephalus lunaris* by a genetic analysis, and high levels of TTX were determined by chemical analysis [81].

On the other hand, eleven members of a family from Duque de Caxias city in Rio de Janeiro were intoxicated by ingestion of *Lagocephalus* pufferfish meat. Neuromuscular symptoms appearing 20 min after ingestion and three patients (two children and one adult) were seriously affected. No deaths were registered and the patients did not present sequelae after the episode [82].

#### 4.2.2. Oceania

A 4-year-old boy was bitten by a blue-ringed octopus (*Hapalochlaena* sp*.*) in a popular beach in Queensland, Australia. After ten minutes of the bite, he presented TTX intoxication symptoms such as vomiting, lost the ability to stand and complained of blurred vision. Twenty minutes later he had acute and progressive skeletal muscle weakness, and was intubated, ventilated, and transferred to a pediatric intensive care unit for specialized supportive care. He was ventilated for a total of 17 h with spontaneous muscular activity returning at around 15 h from envenomation [83].

Other investigations into a series of dog poisonings were carried out on beaches in Auckland, North Island, New Zealand, and resulted in the identification of TTX in the grey side-gilled sea slug, *Pleurobranchaea maculate* [84]. In two of the dog poisoning cases, vomit and gastrointestinal contents were found to contain TTX. Tests for other marine toxins were negative.

#### 4.2.3. Europe

In October 2007, a 49-year-old man was intoxicated in Malaga, Spain, due to the ingestion of the trumpet shell *Charonia lampas lampas*, caught in the southern Portuguese waters. Minutes after the ingestion, the TTX intoxication began (abdominal pain with nausea and vomiting, weakness, difficulty articulating words and keeping the eyelids open, and difficulty breathing). After 72 h these symptoms reversed [85]. The chemical analysis of the trumpet shell demonstrated the presence of TTX in the mollusc [86]. More recently, in 2012, TTX was detected in several bivalves from Vistonikos Bay-Lagos, in Greece during an official shellfish control for the presence of marine biotoxins [59].

Table 3 shows some incidents occurred around the world due to the consumption of food contaminated with TTX, indicating the amount of TTX ingested or the toxin concentration found in food.

**Table 3:** Cases of TTX intoxication during last three decades.

Cases	Species Implicated	Country/Region Contaminated Shellfish	Date	Amount of Toxin or Fish Ingested	Symptoms Started/Deaths	Reference
1	*Charonia sauliae*	Shimizu, Shizuoka, Japan	December 1979	17,000 MU	30 min after ingestion. Fully recovered in 5 days	[77]
1	*Diodon hystrix*	Hawaii, USA	1986	NR	He recovered within 1 week	[79]
3	fugu (pufferfish) brought from Japan	California, USA	April 1996	a middle-quarter of fugu	2–20 min after ingestion	[87]
1	*Takifugu poecilonotus*	Nagasaki, Japan	October 1996	10,000 MU	30 to 60 min after ingestion. He died during the following hour.	[78]
4	NR	Nosy Be, Madagascar	July 1998	16 MU/g	NR	[88]
5	*Takifugu niphoble*	Chungua, Taiwan	January 2000	11 g of the cooked fish liver (280 MU/g)	NR	[67]
6	NR	Taiwan Strait	April 2001	NR	2 to 3 h after ingestion	[89]
6	*Nassarius glans*	Tungsa Island, Taiwan	April 2004	digestive gland (2048 MU/g) and muscle (2992 MU/g)	NR	[90]
202	eight strains of *Shewanella* spp.	China	September 2007	NR	1 to 4 h after ingestion	[91]
156	13 species of pufferfish	Bangladesh	1998–2008	NR	10 min–15 h after ingestion. 40 deaths	[76]
NR	*Lagocephalus inermis*	Nagasaki, Japan	October 2008	residual liver sample showed toxicity as high as 1230 MU/g	NR	[92]
2	Octopus *Hapalochlaena fasciata*	Taipei, Taiwan	December 2010	The concentration of TTX was 31.8–94.3 μg/g (39.1–83.4 ng/mL in the urine and <0.1 ng/mL in plasma).	The symptoms subsided within five days and the patient fully recovered.	[93]
26	*Amoyacaninus* and *Yongeichthys nebulosus*	Guangdong, China	March 2012	100–300 g of fish consumed. The amount of TTX found in muscle and viscera was 9.69 MU/g and 10.42 MU/g in the case of *A. canicus*, and 14.51 MU/g and 15.47 MU/g in the case of *Y. nebulosus*	NR	[94]
12	*Lagocephalus sceleratus*	coast of Reunion Island (Southwest Indian Ocean)	September 2013	NR	fully recovered within a few days	[95]
2	*Lagocephalus lunaris*	Minneapolis, Minnesota	June 2014	5.7–72.3 ppm	Thirty minutes after consumption. After 6 h his symptoms improved	[81]
71	*Carcinoscorpius rotundicauda*	Chon Buri, Thailand	NR	NR	Nineteen patients required artificial ventilation and there were two deaths.	[96]

MU: mouse unit; NR: not reported.

## 5. Therapies for TTX Intoxication

Notwithstanding that in 1984 a work group tried to make use of an anti-cholinesterase drug for treating TTX poisoning, no known antidote exists nowadays [97].

Several investigations have been carried out in order to develop possible treatments against TTX intoxication. In 1989, two anti-TTX antibodies were isolated. These specific antibodies recognized TTX but not the related sodium channel blocker, STX, as determined by competition ELISA [98].

Later in 1995, studies simulating oral intoxication were performed. During these investigations, mice were fed with a lethal dose of TTX by gavage in a suspension of non-fat dry milk in phosphate-buffered saline. Death occurred within 25–35 min in 6/6 mice. However, 500 µg of a new monoclonal antibody isolated during the study, T20G10, administered via the tail vein 10–15 min after oral TTX exposure, prevented death in all cases [99]. On the other hand, other monoclonal antibody isolated in the same year neutralized TTX *in vivo* in other study where intoxicated animals were injected with 100 µg IgG through the tail vein showing 100% survival [100]. Others have made different progress including administrations of monoclonal antibody or antiserum to TTX capable of passively protecting mice from lethal dose of TTX poisoning before or after TTX exposure although these might be still unsatisfactory for actual application [101,102,103,104]. Moreover, an efficacious TTX-experimental vaccine that could protect animals from intraperitoneal challenges of TTX was also developed [105,106,107].

Despite all of these investigations, providing the victim with respiratory support or mechanically ventilation until the TTX is excreted completely, or gastric lavage, are the only treatments available for TTX intoxication and have been shown to reduce deaths [26,74,108,109].

## 6. Medical Applications of Tetrodotoxin

The use of TTX holds on their mechanisms of action, mainly the blockade of Voltage-Gated Sodium Channels (VGSCs), and hence, the alteration of neuronal function. Indeed, it has been shown to exert blockade of the l-type Ca^2+^ current in canine cardiac cells [110]. The limitations for its use are related to their toxic effects, which have been reviewed above; nevertheless, its potent analgesic activity shown in several animal models supports the rationale for its use with therapeutic purposes. On the other hand, it has been proposed that TTX do not pose a genotoxic risk to patients [111], which is an advantage in regards to its use as a drug in humans. There are several examples of potent natural toxins being used as drugs in human medicine [112,113]; for example, botulinum toxin, from the bacterium *Clostridium botulinum*, employed in situations where excessive muscle contraction is observed, or even with cosmetic purposes [114,115]; conotoxin, a synthetic analogue of the cone snail *Conus magnus* peptide ω-conotoxin, for the treatment of severe chronic pain [111] and several analogues have been proposed to have cardioprotective effects, to be useful in Parkinson’s disease, Alzheimer’s disease, and nicotine addiction treatment [116]. Other biotoxins from marine origin, such as those from the STX group, have been proposed for medical applications [117,118].

The most promising therapeutic use of TTX is perhaps in the treatment of certain pains. In fact, before the discovery of TTX, globefish was used in Japanese folk medicine in the treatment of leprosy because globefish flesh alleviated the neuralgia of patients affected with leprosy. After its isolation by Dr. Yoshizumi Tahara from aqueous extract of globe fish ovaries, TTX extracts prepared by Tahara’s method were used to treat neuralgia due to leprosy and to reduce muscle spasms due to tetanus in the early XX century. Also, TTX was given to patients with rheumatoid arthritis due to its analgesic effect. According to Professor Tsuda and Dr. Kawamura, who improved the purification method in 1952, the TTX preparation manufactured by Tahara’s method was not very good in terms of purity, since their crystalline TTX had a LD50 of 4–6 μg/kg mouse and the LD50 of the extract obtained with Tahara’s method was 4–5 mg/kg mouse [1].

We can understand pain as a defensive reaction of the body intended to warn us of different hazards or harms that should be avoided or treated. In this sense, pain “is essential for maintaining bodily integrity and is associated with noxious stimuli, and is therefore called nociceptive pain” [119]. Nociceptive pain is not viewed as a clinical problem and, in fact there are several illness characterized by absence of pain in response to different painful stimuli, such as Congenital Insensitivity to Pain with Anhidrosis (CIPA), an autosomal-recessive disorder resulting from defective neural crest differentiation with loss of the first-order afferent system, which is responsible for pain and temperature sensation [120]. Under certain conditions, this nociceptive pain changes into neuropathic pain, occurring with an abnormally functioning somatosensory nervous system. In other cases, chronic diseases such as cancer, osteo- and rheumatoid arthritis, operations and injuries, and spinal problems, lead to chronic pain [119]. VGSCs play a key role in pain, and TTX-sensitive subtypes have been strongly implicated in normal and pathological pain. Since TTX blocks this subset of VGSCs in a highly selective manner, this agent may have a potential role in relieving pain. TTX binds to a neurotoxin receptor site on the α-subunit of the VGSC at the outer vestibule of the channel and blocks the entry of Na^+^ [121,122]. This blockade inhibits the propagation of action potentials, and hence, blocks impulse conduction in nerves [123].

The response of different organs or tissues to TTX will vary depending on the VGSC isoforms present in their cells, since the response of the different nine existing isoforms varies in kinetics and sensitivity to TTX [113,119,124]. For instance, TTX sensitivity of the cardiac Na^+^ current is different among distinct groups of vertebrates, being more TTX resistant in mammals and reptiles (and some fish like lamprey) than in teleost fish, frog and bird [125]. VGSCs play a key role in nociception, since they are implicated in driving the information to the central nervous system. Dysfunctional VGSCs have been related to several pain states, and data from human genetic studies and transgenic mouse models suggest that specific VGSC are associated with specific types of pain [119,126]. This situation would allow the development of drugs that selectively block a single channel or selected channels and theoretically, the use of specific blockers could help to avoid some adverse effects associated with non-selective sodium channel blockers. Distribution of the diverse isoforms along the body tissues and their implication in different types of pain has been reviewed by Nieto *et al.* [119]. The effects of TTX on acute and on inflammatory pain have not been broadly studied, but it seems that TTX exerts little effect on acute pain. On the other hand, promising results have been achieved against inflammatory pain and even on the neurogenic inflammatory response to an injury [119]. The role of TTX in neuropatic pain has been investigated more intensely than acute or inflammatory pain. TTX has been studied in several animal models, mainly in rodents, but also in rabbits, cats and dogs [40,119,126]. Pain models include cold pain, mechanical pressure, inflammatory pain, heat, visceral pain, pain induced as side effect by therapeutic drugs, formalin test or neuropathic pain Writhing test. Studies of TTX in animal pain models have been recently reviewed [119].

The use of TTX has been investigated for medical purposes other than pain mitigation in animal models. These investigations include several urinary bladder dysfunction studies in pigs [127], treatment of drug addiction in rats [128], corneal injury induced photophobia in rats [129] or schizophrenia in rats [130].

Some researchers are trying to make use of the analgesic activity of TTX to treat various types of pains such as in severe cancer [29,34,119]. In a clinic trial performed in Canada, TTX was administered subcutaneously to cancer patients. The time course of the apparent analgesic response to TTX was an additive analgesic effect until Day 4 or 5, the effect peaked around Day 10, and then became less after that time, wearing off two weeks or longer after TTX was first administered. During the study, physical examination, vital signs, oxygen saturation, corrected QT interval (QTc) and other electrocardiographic parameters, neurological examination, clinical chemistry, and haematology measures were not affected by TTX, although three TTX-treated patients were withdrawn from the study due to adverse effects occurrence (moderately severe but transient ataxia, malignant epidural spinal cord compression and transient moderate dysphagia, respectively). Overall, treatment-emergent adverse events in TTX-treated patients were mild and related to tingling, numbness, or other transient sensory symptoms [29]. In a later clinic trial, the patients participating in the former enrolled a study designed to evaluate long-term TTX safety and efficacy. In this study, 30 μg TTX was administered subcutaneously twice daily for four days. One patient was withdrawn because of adverse effects. Toxicity was usually mild or moderate, and remained so through subsequent treatment cycles, with no evidence of cumulative toxicity or tolerance with long-term administration. Surprisingly, only about 50% of patients responded to TTX, with no explanation for this achievement.

TTX is in fact in Phase III trials as an agent (Tectin^®^) against inadequately controlled pain related to cancer by WEX Pharmaceuticals in the USA, together with a Phase II trial under the same company, again in the USA, against the neuropathic pain resulting from chemotherapy-induced peripheral neuropathy [131]. Tectin^®^ contains TTX at a concentration of 15 μg/mL (47 μM), in 2 mL ampoules for subcutaneous injection [123]. A low intramuscular dose (10 μg TTX twice daily, but not 5 μg TTX twice daily) has also been shown to help in reducing cue-induced increases in heroin craving and associated anxiety [32]. Also, it has been employed in alleviating acute heroin withdrawal syndrome at dosages of 5 and 10 μg administered three times a day with low side effects [132] and it has been employed in heroin and morphine addiction animal models [32]. TTX directed to management of opiate withdrawal symptoms (Tetrodin TM) started Phase IIa clinical trials in Canada and a formulation intended for local anesthesia (Tocudin TM) started preclinical studies [133]; taking advantage that, on the contrary to other local anaesthetics, TTX does not cause direct myocardial depression, and they cross the blood brain barrier very poorly, reducing their risk of seizures or central nervous system depression [123].

VGSCs have been proposed as therapeutic targets for different neurological syndromes related to disorders in neuronal excitability, such as epilepsy, migraine, neurodegenerative diseases [134]. US patent US20140221286 A1 claims for the use of sodium channel blockers for the treatment of hyperglycemia based on the discovery that sodium-channel blockers inhibit the secretion of glucagon from pancreatic alpha cells [135]. For all these indications, TTX applicability would depend on the equilibrium among therapeutic and toxic doses.

An extended use of TTX would depend on the availability of the molecule independently on the natural sources, but this would not be a problem since its chemical synthesis has been already achieved [136,137,138].

## 7. Conclusions

Tetrodotoxin is a ubiquitous toxin which has reached both terrestrial and aquatic environments and different taxonomic groups, from bacteria to vertebrates. Despite its mechanism of action and molecular target in humans is well known, a specific treatment for tetrodotoxin food poisoning has not been achieved yet. The blockade of different VGSCs makes this toxin a promising tool as therapeutic drug, especially for pain treatment.
